# Additional C-type lectin receptors mediate interactions with *Pneumocystis* organisms and major surface glycoprotein

**DOI:** 10.1099/jmm.0.001470

**Published:** 2021-12-10

**Authors:** Theodore J. Kottom, Eva M. Carmona, Kyle Schaefbauer, Andrew H. Limper

**Affiliations:** ^1^​ Thoracic Diseases Research Unit, Departments of Medicine and Biochemistry, Mayo Clinic College of Medicine, Rochester, MN, USA

**Keywords:** pneumocystis, C-type lectin receptors (CLRs), carbohydrate recognition domains (CRDs), major surface glycoprotein (Msg)

## Abstract

**Introduction:**

Pathogen-associated molecular patterns’ (PAMPs) are microbial signatures that are recognized by host myeloid C-type lectin receptors (CLRs). These CLRs interact with micro-organisms via their carbohydrate recognition domains (CRDs) and engage signalling pathways within the cell resulting in pro-inflammatory and microbicidal responses.

**Hypothesis/Gap statement:**

In this article, we extend our laboratory study of additional CLRs that recognize fungal ligands against *Pneumocystis murina* and *Pneumocystis carinii* and their purified major surface glycoproteins (Msgs).

**Aim:**

To study the potential of newly synthesized hFc-CLR fusions on binding to *Pneumocystis* and its Msg.

**Methods:**

A library of new synthesized hFc-CLR fusions was screened against *Pneumocystis murina* and *Pneumocystis carinii* organisms and their purified major surface glycoproteins (Msgs) found on the respective fungi via modified ELISA. Immunofluorescence assay (IFA) was implemented and quantified to verify results. mRNA expression analysis by quantitative PCR (q-PCR) was employed to detect respective CLRs found to bind fungal organisms in the ELISA and determine their expression levels in the mouse immunosuppressed Pneumocystis pneumonia (PCP) model.

**Results:**

We detected a number of the CLR hFc-fusions displayed significant binding with *P. murina* and *P. carinii* organisms, and similarly to their respective Msgs. Significant organism and Msg binding was observed for CLR members C-type lectin domain family 12 member A (CLEC12A), Langerin, macrophage galactose-type lectin-1 (MGL-1), and specific intracellular adhesion molecule-3 grabbing non-integrin homologue-related 3 (SIGNR3). Immunofluorescence assay (IFA) with the respective CLR hFc-fusions against whole *P. murina* life forms corroborated these findings. Lastly, we surveyed the mRNA expression profiles of the respective CLRs tested above in the mouse immunosuppressed Pneumocystis pneumonia (PCP) model and determined that macrophage galactose type C-type lectin (*Mgl-1*), implicated in recognizing terminal N-acetylgalactosamine (GalNAc) found in the glycoproteins of microbial pathogens was significantly up-regulated during infection.

**Conclusion:**

The data herein add to the growing list of CLRs recognizing *Pneumocystis* and provide insights for further study of organism/host immune cell interactions.

## Introduction

*Pneumocystis* spp. cause *Pneumocystis* pneumonia (PCP) in mammalian hosts, and in humans *Pneumocystis jirovecii* pneumonia (PJP). *Pneumocystis* possess two dominant life forms including the ascus (cyst), which contains β−1,3 and β−1,6 linked glucans, as well as surface-associated glycoprotein, termed major surface glycoprotein (Msg), and the more prevalent and diminutive trophic forms, which bears abundant amounts of Msg [1–3]. In previous studies, the authors and others have shown central roles of certain C-type lectin receptors (CLRs) in pro-inflammatory responses to *Pneumocystis*. Examples include CLRs Dectin-1 and macrophage-inducible C-type lectin (Mincle), where deletion of the respective genes results in significant increases in organism burden and subsequent mortality [4, 5]. In contrast, absence of Dectin-2 or the mannose receptor (MR) had no effect on organism burden, even though their deletion led to defective proinflammatory cytokine production and migration of phagocytic cells during PCP, respectively [6, 7]. CLRs also have the ability to ‘cross-talk’ with one another, leading to either increased or decreased proinflammatory responses, respectively [8–10]. Therefore, simple, single deletions of one CLR in a pathogenic fungal model, although informative, may not represent the full picture of host recognition and immune response to the organism during infection. In prior studies, we have screened and demonstrated that a number of previously implicated CLR-CRDs such as Dendritic Cell-Specific Intercellular adhesion molecule-3-Grabbing Non-integrin (DC-SIGN), Dectin-2, Macrophage C-type lectin (MCL), MR, and Mincle known to be important in host fungal interactions, also interacted with *Pneumocystis* to a significant degree [11]. In this study, we continue our screening of CLRs with a panel of newly synthesized hFc-fusion proteins against *Pneumocystis murina* and *Pneumocystis carinii* organisms and their respective isolated Msgs. In addition, immunofluorescence microscopy was implemented to verify CLR hFc-fusion binding to *P. murina*. Lastly, we surveyed the mRNA transcriptional profile in the mouse immunosuppressed PCP model of the most significant CLRs discovered in our initial solid-state CLR screening and report those results. Our data provide valuable quantitative and qualitative analysis of additional CLRs in *Pneumocystis* host immune recognition.

## Methods

### Animals

All animal experiments were conducted in accordance with the guidance of the Mayo Institutional Animal Care and Use Committee. *P. murina* pneumonia was induced in mice immunosuppressed with GK 1.5 monoclonal antibody as previously described [12]. The GK1.5 antibody was obtained from Bio X Cell (Lebanon NH). Briefly, mice were given two intraperitoneal injections of 0.3 mg in 0.2 ml over the first week. Subsequently, mice were given weekly injections of the antibody for the 8–10 weeks. Organisms were purified as previously described [13]. The rat *P. carinii* pneumonia model has been described elsewhere [14]. Briefly, immunosuppression in rats was achieved by the addition of dexamethasone (1 mg l^−1^) and tetracycline (500 mg l^−1^) (both Sigma Aldrich) to prevent secondary bacterial infections. After 8–10 weeks of immunosuppression, rats were sacrificed and organism purified as previously described [15]. *Pneumocystis* glycoprotein termed Msg was prepared as characterized as previously reported [6]. Briefly, Msg preparations were run on PAGE gels and assessed by silver staining, demonstrating a distinct single band of the published molecular weight (MW) of Msg. Furthermore, Western blotting with a specific *Pneumocystis* spp. anti-Msg monoclonal antibody (5E12), yielded a specific band at the appropriate MW [15].

### CLR hFc-fusions

CLR human IgG1 Fc fragment (hFc)-fusions hFc control, mCLEC9A, mCLEC12A, mLangerin, myeloid DAP-12-associating lectin (mMDL), mMGL-1, and mSIGNR3 have been described previously [16]. Briefly, RNA from mouse spleen was isolated and converted to cDNA. PCR was then implemented to generate the extracellular region containing the ligand binding domain of the respective CLR. Next, fragments were cloned into pFuse-hIgG1-Fc (human) expression vector (InvivoGen, San Diego, CA) and transiently transfected into CHO-S cells. Fusion proteins were purified after 4 days utilizing HiTrap protein G HP columns (GE Healthcare, Piscataway, NJ). mMCL has been described and utilized by our lab previously and functioned as a positive binding control [11].

### hFc-CLR fusion ELISA

Briefly, live mixed *P. murina* or *P. carinii* (~1×10^6^ life forms) were fixed in 4% paraformaldehyde, 0.1M K_2_HPO_4_, pH 6.5 for 90 min. The organisms were then washed three times with 0.1M K_2_HPO_4_, pH 6.5. These organisms or native *P. carinii* Msg (2.0 µg/well) or native *P. murina* Msg (0.2 ug/well) was plated onto 96-well microtitre plates and incubated at 4 °C overnight. The following day, plates were washed three times with 100 µl PBS-Tween (PBS-T). Next, wells were blocked with PBS/10% FBS/2.5% milk) at 4 °C for 2 h. After three washes with PBS-T, the respective CLR hFc-fusion proteins (200 ng) [16] were added in lectin binding buffer (LBB) (50 mM HEPES, 5 mM MgCl_2_ and 5 mM CaCl_2_) for 2 h at 4 °C. Next, 1 : 5000 dilution of HRP goat anti-human Ig Fc antibody (SouthernBiotech) in blocking buffer was added for 1 h at 4 °C. Lastly, after washing the plates three times with 1X PBS-T, 1X TMB substrate was applied for 20 min at RT, followed by stopping the reaction with 2.0 M H_2_SO_4_. Plates were read in a VERSAmax microplate reader (Molecular Devices) at 450 nm. Three to four independent experiments were conducted in duplicate wells for each assay.

### Immunofluorescence studies

Briefly, mixed *P. murina* life forms were prepared as above for the solid-state ELISA. Organisms were next applied to poly-l-lysine coated slides for 15 min at RT. Slides were then washed with 1X PBS and incubated with 500 ng of the respective hFc-CLR fusion or hFc alone in LBB at 4 °C overnight. After washing three times in LBB, samples were incubated for 2 h with 1 : 250 Alexa Fluor 488 goat anti-human IgG (H+L) conjugated antibody (Life Technologies) at 4 °C. Finally, the slides were washed three times with 1X PBS, cover slips applied and viewed on an Olympus BX53 fluorescence microscope.

### CLR mRNA expression during *P. murina* PCP model

Lungs were harvested after 10 weeks of *P. murina* infection and tissue samples (30 mg) were homogenized using a TissueLyser LT (Qiagen) at 50 oscillations/s for 5 min. Total RNA was isolated, and an aliquot (200 ng) was used to generate cDNA. Steady-state mRNA expression of the respective CLRs in these samples was determined by quantitative PCR (qPCR) analysis conducted with the respective primer sets (Table 1) and was expressed as normalized to Beta-2 microglobulin (*B2m*) or glyceraldehyde-3-phosphate dehydrogenase (*Gapdh*) to verify equal cDNA contents.

**Table 1.** PCR primers used in this study

Gene name	Forward primer	Reverse primer
*Clec12A*	ACCATGTCCAAAGGGTTCAG	AGTGGATATTGTGTGCGATCTT
*Langerin*	GTTCTGAGGAAACCTCTCTGTATC	CACACGACCTCTTTCAGTCTT
*Mcl*	TCAGACTACCACACGAGAGTAA	TCAGCAAGTCCCAGGAAATAAG
*Mgl-1*	GAACTCAAGGATCGAGGAGAAA	CTTTAGACAACACCACCTCCA
*Signr3*	GACTGATGAGGAGCAGACTTTC	GGATGGCTGGAATGATCTCAG
*B2M*	CTCGGTGACCCTGGTCTTTC	GGATTTCAATGTGAGGCGGG
*Gapdh*	TTGCCATCAACGACCCCTTC	ACTCCACGACATACTCAGC

### Statistical methods

All data are presented as mean+/-sd or sem. Statistical testing was conducted between CLR hFc-fusions and the hFc control. Differences between conditions were first evaluated using ANOVA and subsequently unpaired Student’s *t*-tests were performed on data sets. Statistics were performed using GraphPad Prism version 9.2.0 software, and differences were considered to be statistically significant at *P*<0.05.

## Results

### CLR ELISA binding patterns

**Fig. 1.**
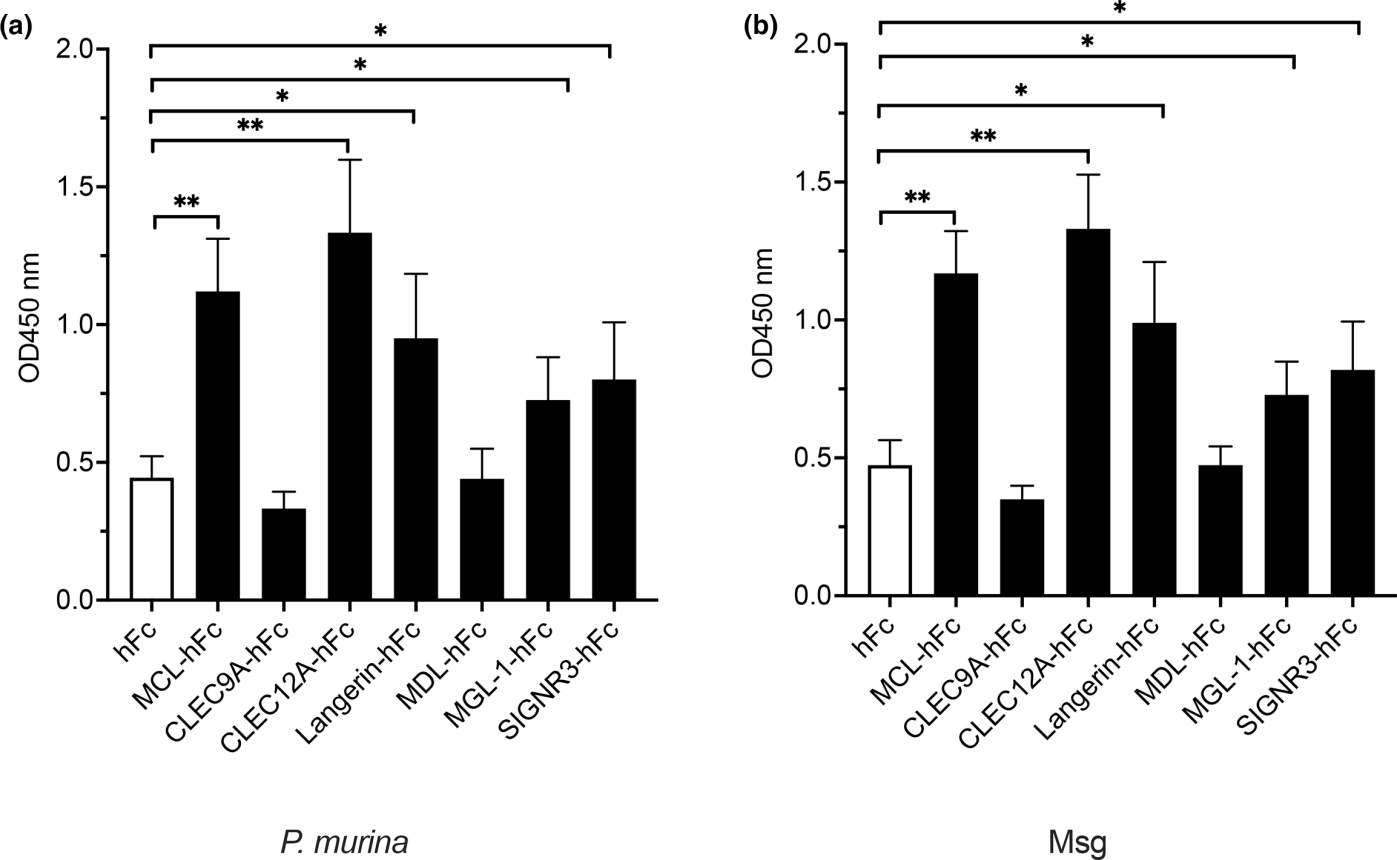
Binding of respective CRD Fc-fusion protein to *P. murina* organisms and *P. murina* major surface glycoprotein (Msg) as measured by absorbance at 450 nm. Total *P. murina* organisms or *P. murina* isolated Msg were applied to 96-well microtitre plates and probed with the respective hFc-fusion protein. *P<0.05, **P<0.005.

As previously described by our group [11], we implemented an ELISA-based method with a library of newly designed CLR hFc-fusion proteins against *P. murina* and *P. carinii* life forms and the organisms' abundant surface glycoproteins, Msgs. Using human hFc protein as a negative control, and the previously known interaction of *Pneumocystis* with CLR MCL as a positive control [11] we determined that CLEC12A, Langerin, MGL-1 and SIGNR3 displayed significant binding to *P. murina* and *P. carinii* and their respective Msgs (Figs 1a, b and S1a, b, available in the online version of this article).

### Correlation between ELISA and immunofluorescence results

**Fig. 2.**
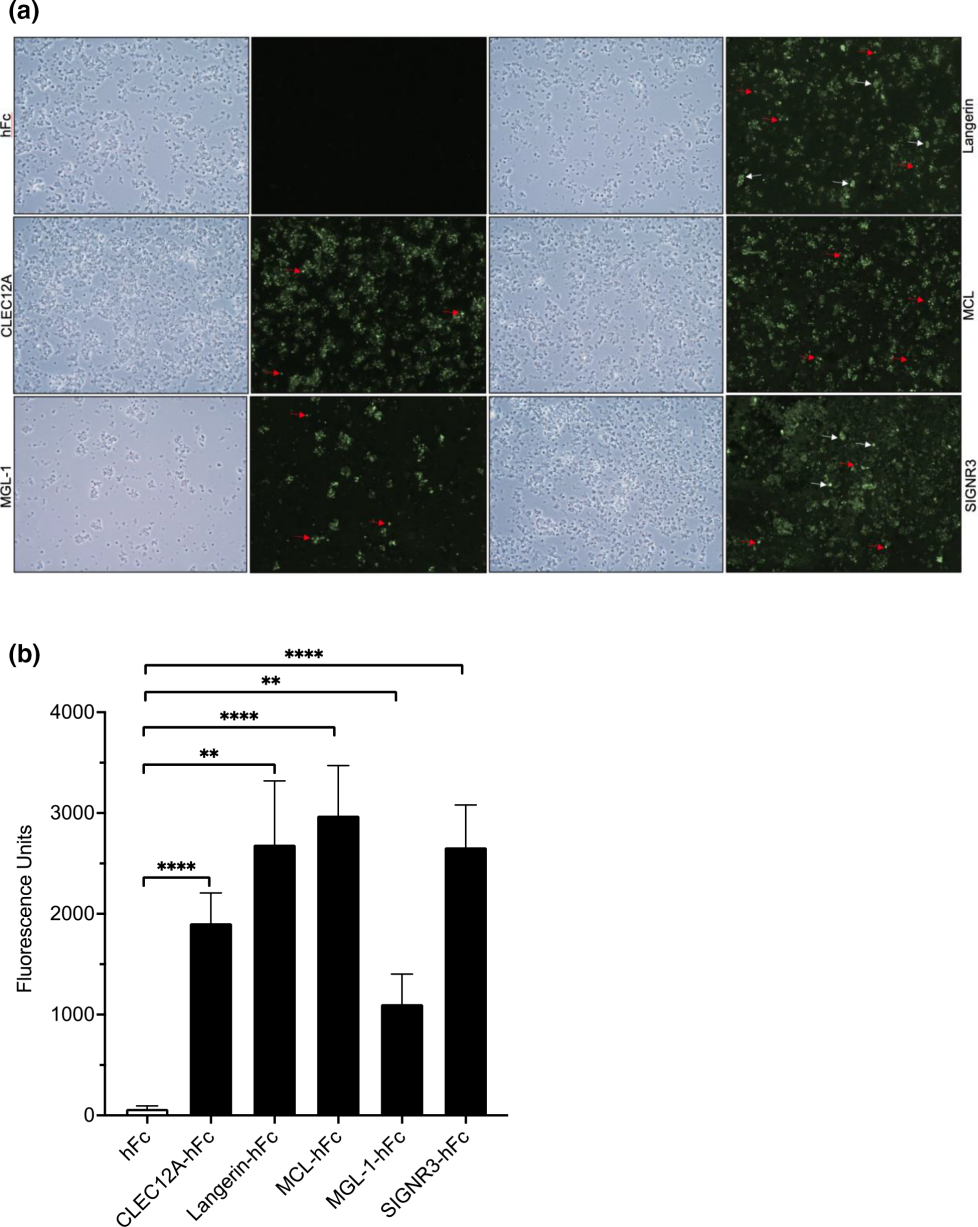
(a) Soluble CLR hFc-fusions bind *P. murina* life forms as visualized by microscopy. (Left panels) Phase-microscopy image of *P. murina* life forms. (Right panels) *P. murina* organisms were stained with the Fc fragment alone or the respective CLR hFc-fusion, followed by staining and viewing with FITC-conjugated anti-human Fc antibody at 25× magnification. White arrows indicate cyst life forms, whereas red arrows indicate trophic life forms. (b) Bar graph of seven random similar sized rectangle fields of photos of the respective CLR hFc-fusions binding to *P. murina* life forms and graphed as fluorescence units. Analysis conducted with LI-COR Image Studio (version 5.2.5) software. *P<0.05, **P<0.005, ****P<0.0001.

Immunofluorescence assay (IFA) of the CLR hFc panel tested against immobilized *P. murina* life forms was conducted next. Visualization of these results confirmed the solid-state ELISA experiments, notably the respective CLR hFc-fusion panel bound *P. murina* with different fluorescent intensities as well as binding to different life forms present on the slides. No appreciable binding was observed for hFc, whereas the positive control MCL bound *P. murina* organisms similarly to our previously published data [11]. Our observations reveal that Langerin and SIGNR3 hFc-fusions bind both the cyst (white arrows) and trophic (red arrows) life forms similarly, whereas CLEC12A and MGL-1 hFc-fusions bind trophic forms with a high degree of selectivity, similar to the positive MCL control (Fig. 2). Fig. 2b shows the quantification of the fluorescence in graph form and also demonstrates that all the respective CLR fusions tested bind *P. murina* organisms with significance, similar to our solid-state ELISA binding assays above. The possible role and importance of all four of these CLRs are currently uncharacterized in PCP, and therefore data presented here provides an initial starting point for future studies to determine their roles in host immune recognition and downstream inflammatory signalling during PCP.

### Enhanced mRNA expression of *Mgl-1* RNA CLR in the PCP model

**Fig. 3.**
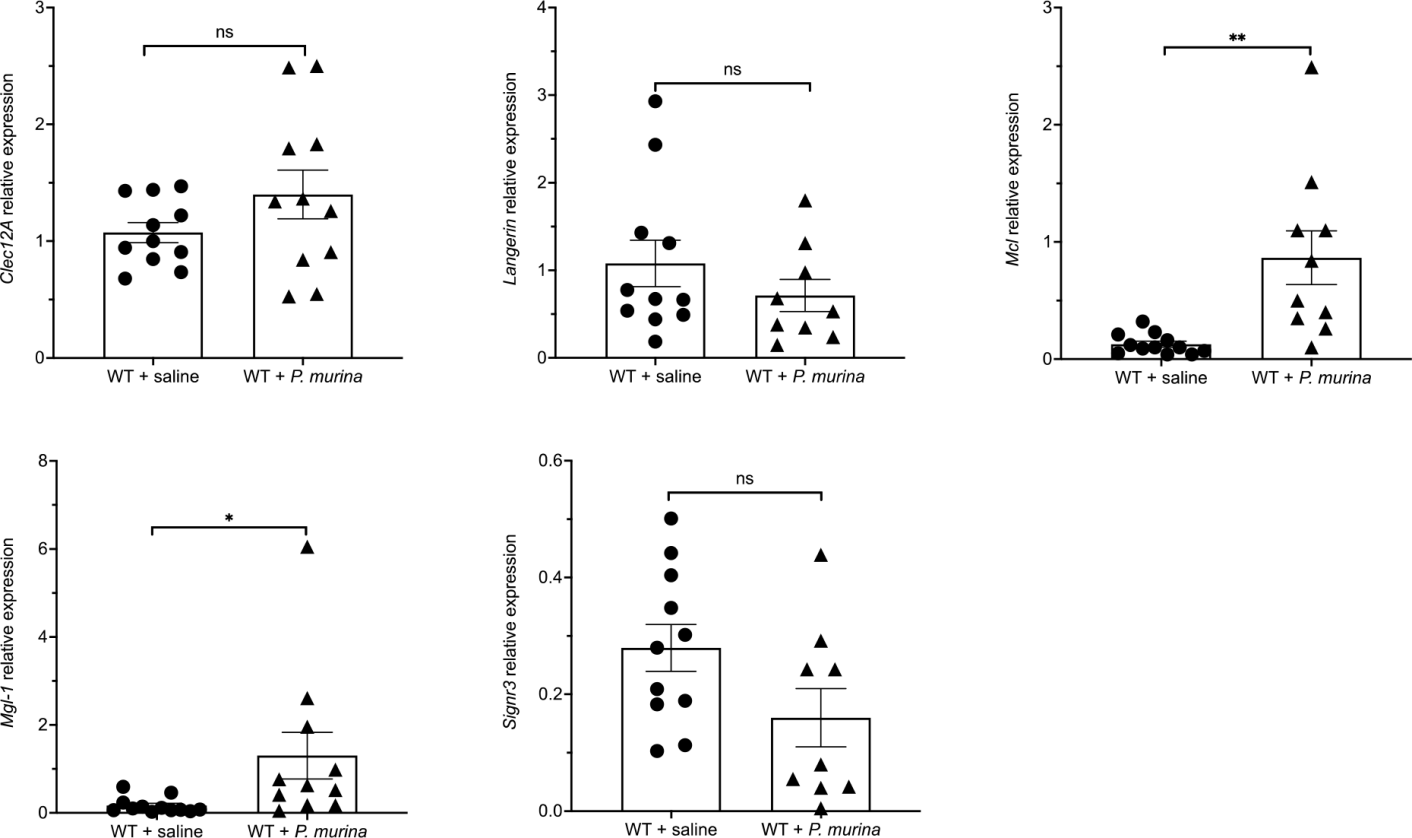
The expression of the respective CLRs during PCP. The mRNA expression levels of *Clec12A*, *Langerin*, *Mgl-1* and *Signr*3 were determined in the animal infection model after 10 weeks of infection. *Mcl* was used as a positive control. The mRNA levels were quantified by qPCR and beta-2 microglobulin (*B2m*) used as a reference gene. A total of 9–11 mice were used per group tested. *P<0.05, **P<0.005.

Previous studies have indicated that CLR Mincle mRNA is elevated to a significant degree in *P. murina* infected mouse lung tissue compared with normal uninfected lung RNA. This observation was also noted in macrophages infected with bacteria *in vitro* as well as in bacterial and fungal pneumonia models. These data suggest a known importance for at least in part in these respective infection models [4, 17–20]. Therefore, in parallel fashion, we evaluated whether overall mRNA expression was increased in a mouse PCP infection model of the four CLRs of interest. Notably, and as confirmation of our previous findings [11], *Mcl* was significantly upregulated in the PCP rodent model. Of the four newly identified CLRs in *Pneumocystis* interactions, we determined *Mgl-1* mRNA was enhanced to a significant degree in *P. murina* infected lung tissue compared with normal uninfected lung RNA using *B2m* (Fig. 3) as well as *Gapdh* (Fig. S2) as reference genes.

## Discussion

Innate immune receptors bind fungi primarily through the carbohydrates embedding or lining the organism cell wall. The prototypic fungal cell wall is composed of chitin, β−1,3 and β−1,6 glucans, α-glucans, as well as a variety of mannoproteins [21]. Binding of these ligands via these pattern recognition receptors (PRRs) provide initial frontline defence and subsequent downstream modulation upon contact with micro-organisms [22]. Currently, there are more PRRs for fungi than any other type of organisms [23]. Previously, we and others have shown a number of CLRs important for host immune response to *Pneumocystis*. These CLRs include Dectin-1, Dectin-2, Mincle, MR and DC-SIGN [4–6, 24, 25].

In this study we also demonstrated that all the CLRs that bound to *Pneumocystis* organisms bound *P. carinii* and *P. murina* organisms and their respective Msgs in similar fashion. The CLRs herein, typically recognize carbohydrate structure, which is much more conserved in fungi than, for example, their respective proteins [26]. We also have shown previously that the mouse CLR Dectin-2 can indeed bind isolated Msgs from both *P. carinii* and *P. murina* [6]. Therefore, most likely, mannan and glucan structures in *Pneumocystis* species have similar degrees of conservation and therefore account for the similar binding results we are seeing of these CLRs tested in both *P. carinii* and *P. murina*.

Looking at the CLRs in sequential order, CLEC12A an inhibitory CLR, contains an immunoreceptor tyrosine-based inhibitory (ITIM) motif in its cytoplasmic tail that can associate with SHP-1 and SHP-2, both signalling phosphatases [27]. Originally described as a uric acid crystal negative regulatory receptor involved in controlling noninfectious inflammation [28], recent studies also suggest a role in recognizing plasmodial hemozoin [29]. Hemozoin is a by-product of blood digestion from blood feeding parasites, such as malaria parasites caused by *Plasmodium* spp. Mice deficient in CLEC12A were protected from experimental cerebral malaria (ECM) [29]. Hemozoin is considered a viable drug target due to its requirement for malaria parasite survival and absence from the mammalian host. Anti-malarial drugs such as chloroquine bind hemozoin and block the addition of new haem groups to the growing crystal structure [30]. Although no known hemozoin-like crystal structures are known in *Pneumocystis*, researchers have demonstrated previously that chloroquine and analogues indeed have anti-*Pneumocystis* activity [31]. Further studies are needed to determine the *Pneumocystis* ligand(s) for CLEC12A and also its potential role in PCP.

Next, we observed significant binding of Langerin to both *Pneumocystis* life forms and Msg via the ELISA binding assay. Similarly, by IFA, Langerin CLR appears to bind both cystic and trophic forms. Langerin (CD207) is expressed exclusively by a subset of dendritic cells known as Langerhans cells [32]. Present in mucosal epithelium, they play an important role in invading microbial pathogens by binding antigens and migrating to draining lymph nodes to present antigens to T cells [33]. A number of pathogens including bacterial, viral and fungal are known to interact with Langerin [34]. From a fungal context, numerous studies have shown that the CLR binds to fungal mannose, GlcNAc and β-glucans from a number of pathogenic fungi [34]. Therefore, our results are as expected since *Pneumocystis* contains abundant β-glucan carbohydrate (cyst forms) as well as mannose containing protein such as Msg (trophic forms). The use of available selective Langerin DTR (diphtheria toxin receptor) mice to deplete Langerin cells would be of valuable interest in determining the role of this cell type and receptor in the rodent PCP model [35].

The third CLR tested in this study that displayed significant binding was MGL-1. In mice there are two homologues of human MGL, MGL-1 and MGL-2, respectively. MGL in humans is expressed *in vivo* by DCs of the epidermis and lymph nodes, and *in vitro* of bone-marrow derived macrophages (BMDMs) [36]. MGL has reported to bind exclusively to terminal α- or β *N*-acetylgalactosamine (GalNAc or Tn) sequences [37]. MGL via the GalNAc terminated lipopolysaccharide (LPS) as well as glycoproteins bind bacteria such as *Neisseria gonorrhoeae *. These interactions lead to Th2 lineage via IL-4 production [38]. Mouse MGL-1 can recognize *N*-GalNAc and galactose [39]. Interestingly, *Pneumocystis* has indeed been shown to contain GalNAc residues [40]. Therefore, the role of the MGL mouse homologue MGL-1 in mouse PCP infection should be examined to determine if like similar CLRs Dectin-1 and Mincle its presence is needed to elicit proper host immune proinflammatory response and control of organism burden in the lung.

Lastly, we show appreciable binding of CLR SIGNR3 to *Pneumocystis* and Msg. SIGNR3 is the closest murine homolog to human DC-SIGN [41]. Previous reports show that this CLR can bind a number of microbial pathogens including *Mycobacterium tuberculosis* and *Candida albicans* [42]. Fungal ligands for SIGNR3 include zymosan and mannan [43]. In our previous CLR screen we demonstrated that human DC-SIGN also binds *Pneumocystis* organisms as well as Msg [11]. These results were also reported by others [25]. Interestingly, it has been shown that although DC-SIGN can indeed bind the fungal organisms, this does not result in downstream activation. Lack of or weak affinity has been proposed as a reason why this occurs [25]. Previous researchers have shown that in the mouse dextran sulphate sodium (DSS)-induced colitis model, SIGNR3 deficiency can lead to exacerbated colitis, as well as an accompanying increase in TNF-alpha production. The authors conclude from their findings that SIGNR3 may be important in maintaining ‘intestinal immune homeostasis’ [43]. The finding that SIGNR3 indeed bind *Pneumocystis* significantly, provides further evidence of a possible role of DC-SIGN in PCP. Future studies should include conducting the PCP model in SIGNR3 knockout mice and determining whether this CLR is important in proper inflammatory response and control of organism burden in the lung.

In our last set of experiments, we examined the mRNA expression of the four respective aforementioned CLRs during PCP. As expected, and reported previously, *Mcl* was significantly upregulated in the 10-week PCP model [4] and served as a positive control for this analysis. Of the four CLRs tested, we determined that *Mgl-1* was also significantly upregulated in this model. Previous reports have shown that other CLRs involved in microbial infections including those with *C. albicans, Malassezia* spp. and *M. tuberculosis* are also upregulated during infection, and this increased upregulation may be important for control of the infection [17–20]. It should be noted that although we only saw significant increased expression of CLR *Mgl-1* in the PCP model, further individual time-point analysis of the other CLRs examined in this study early in the infection might show significant increases in mRNA expression. For example, in mouse lungs infected with *Streptococcus pneumoniae* the levels of Mincle mRNA steadily and significantly increase at 12 and 24 h post-infection but then decrease significantly to near baseline levels by 72 h post-infection suggesting early importance for this CLE in this infection [44]. It may very well be that the CLRs in this study may also display time-course-dependent expression levels as well at different points in PCP that may be critical for the infection. We have also noted increased mRNA expression of CLR Mincle during PCP, a critically important CLR in PCP to help control organism burden and proper inflammatory response [4]. In the future, we plan to determine the importance of MGL-1 by testing the PCP model in MGL-1-deficient mice. Specifically, others have shown that human MGL interacting with CD45 on CD4^+^ T cells can result in T-cell apoptosis leading to dampened inflammatory response and also concurrently activate IL-10 causing inhibition of cell maturation and migration [45]. It will be interesting therefore to determine if MGL-1 is needed in both immunocompetent as well as immunosuppressed models of PCP and importance in establishing or maintaining infection in these respective models.

The data presented here expand on our previous screening of CLR/*Pneumocystis* interactions and provide new possibilities for examining the role of these CLRs in PCP and how these novel binding events may contribute to the pathogenicity of *Pneumocystis* infection.

## Supplementary Data

Supplementary material 1
