# Supplementary material for: Additional C-type lectin receptors mediate interactions with Pneumocystis organisms and major surface glycoprotein
Source: J Med Microbiol. 2021 Dec 10;70(12):001470. doi: 10.1099/jmm.0.001470 (PMC8744274; doi:10.1099/jmm.0.001470)
Supplement: Supplementary material 1 [file jmm-70-1470-s001.pdf]

Supplementary Material

Figure S1A

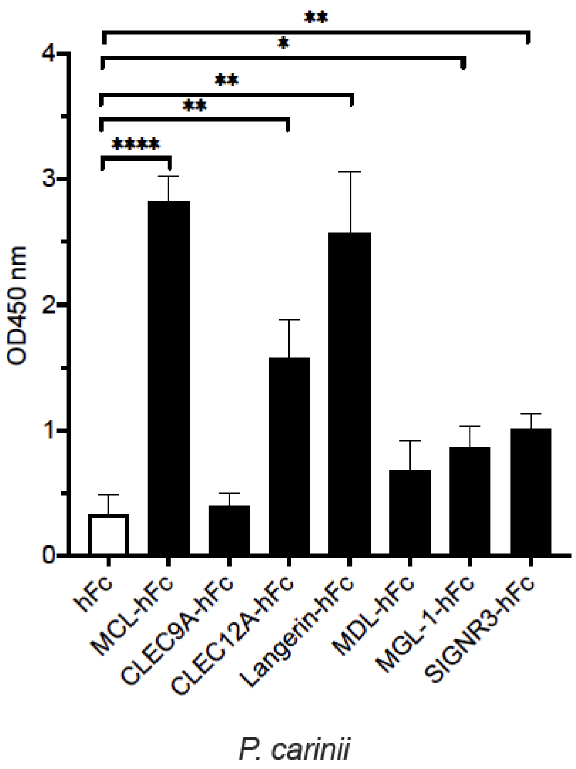

Figure S1B

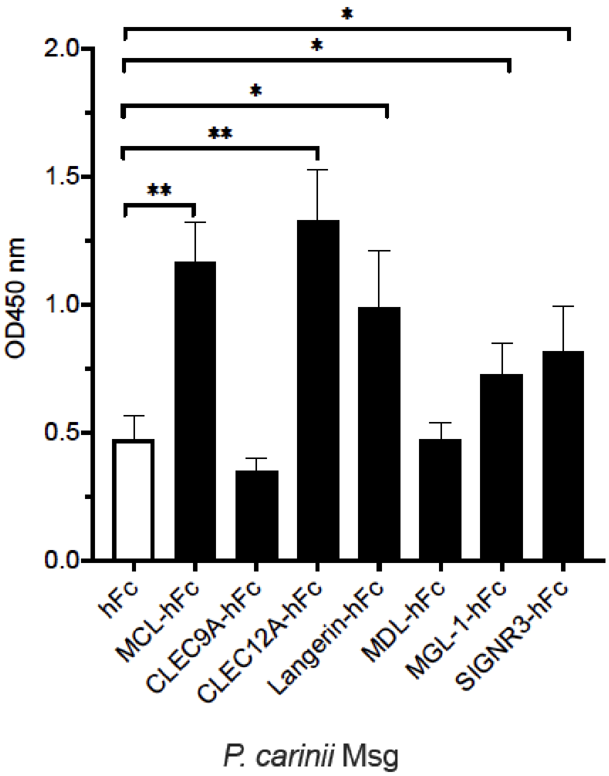

Figure S1A and S1B. Binding of respective CRD Fc-fusion protein to *P. carinii* organisms and *P. carinii* major surface glycoprotein (Msg) as measured by absorbance at 450 nm. Total *P. carinii* organisms or *P. carinii* Msg were applied to 96 well microtiter plates and probed with the respective hFc-fusion protein. *P<0.05, **P<0.005, ****P<0.0001.

Figure S2

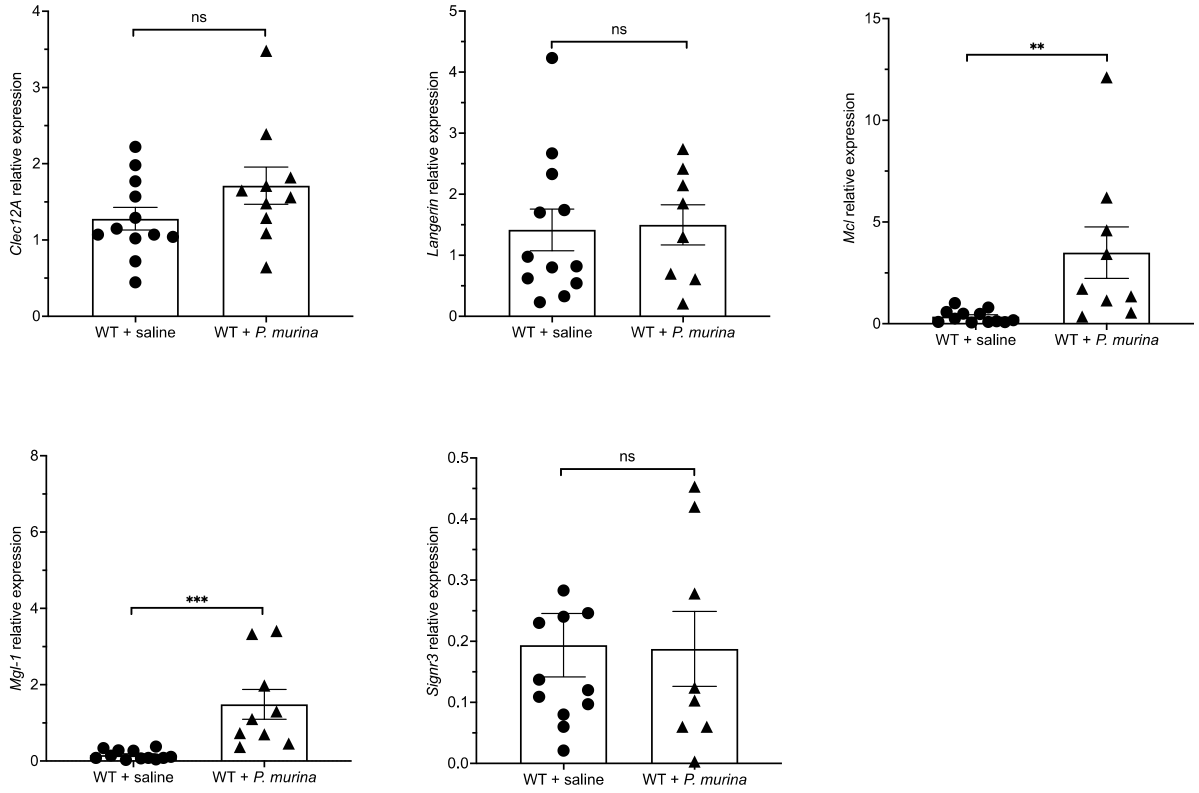

Figure S2. The expression of the respective CLRs during PCP. The mRNA expression levels of *Clec12A*, *Langerin*, *Mgl-1*, and *Signr3* were determined in the animal infection model after 10 weeks of infection. *Mcl* was used as a positive control. The mRNA levels were quantified by qPCR and glyceraldehyde-3-phosphate dehydrogenase (*Gapdh*) used as a reference gene. A total of 8-12 mice were used per group tested. ***P*<0.005, ****P*<0.001.
